# Molecular epidemiological characteristics, variant spectrum and genotype-phenotype correlation of glucose-6-phosphate dehydrogenase deficiency in China: A population-based multicenter study using newborn screening

**DOI:** 10.1371/journal.pone.0310517

**Published:** 2024-10-22

**Authors:** Minyi Tan, Xiulian Liu, Yinhong Zhang, Yifan Yin, Ting Chen, Yulin Li, Lulu Feng, Bo Zhu, Chunjing Xu, Chengfang Tang, Meng Sun, Liyun Jia, Weiwei Jin, Chunna Fan, Hui Huang, Xiaohua Wang, Jizhen Feng, Hui Zou, Lianshu Han, Jingkun Miao, Baosheng Zhu, Cidan Huang, Yonglan Huang

**Affiliations:** 1 Department of Guangzhou Newborn Screening Center, Guangzhou Women and Children’s Medical Center, Guangdong Provincial Clinical Research Center for Child Health, Guangzhou Medical University, Guangzhou, China; 2 Department of Neonatal Disease Screening Center, Hainan Women and Children’s Medical Center, Hainan, Haikou, China; 3 Department of Medical Genetics, The First People’s Hospital of Yunnan Province, National Health Commission Key Laboratory of Preconception Health Birth in Western China, Yunnan Provincial Key Laboratory for Birth Defects and Genetic Diseases, Yunnan Provincial Clinical Research Center for Birth Defects and Rare Diseases, The Affiliated Hospital of Kunming University of Science and Technology, Kunming, Yunnan, China; 4 Department of Pediatrics, Chongqing Health Center for Women and Children &Women and Children’s Hospital of Chongqing Medical University, Chongqing, China; 5 Department of Pediatric Endocrinology and Genetic Metabolism, Shanghai Institute for Pediatric Research, Xinhua Hospital, Shanghai Jiaotong University School of Medicine, Shanghai, China; 6 Department of Neonatal Disease Screening Center, Jinan Maternity and Child Health Hospital affiliated to Shandong First Medical University, Jinan, China; 7 Department of Genetic, Shijiazhuang Maternal and Child Health Hospital, Hebei Shijiazhuang, China; 8 Department of Genetics, Inner Mongolia Maternity and Child Health Care Hospital, Hohhot, Inner Mongolia, Hohhot, China; 9 BGI Genomics, BGI-Shenzhen, Shenzhen, China; 10 Tianjin Medical Laboratory, BGI-Tianjin, BGI-Shenzhen, Tianjin, China; 11 College of Life Sciences, University of Chinese Academy of Sciences, Beijing, China; University of Cape Town Faculty of Science, SOUTH AFRICA

## Abstract

**Background and aims:**

Newborn screening (NBS) for glucose-6-phosphate dehydrogenase (G6PD) deficiency by biochemical tests is being used worldwide, however, the outcomes arising from combined genetic and biochemical tests have not been evaluated. This research aimed to evaluate the outcomes of application of combined genetic and biochemical NBS for G6PD deficiency and to investigate the molecular epidemiological characteristics, variant spectrum, and genotype-phenotype correlation of G6PD deficiency in China.

**Methods:**

A population-based cohort of 29,601 newborns were prospectively recruited from eight NBS centers in China between February 21 and December 30, 2021. Biochemical and genetic NBS was conducted simultaneously.

**Results:**

The overall prevalence of G6PD deficiency was 1.12% (1.86% for male, and 0.33% for female; 1.94% for South China and 0.08% for North China). Genetic NBS identified 10 male patients undetected by biochemical NBS. The overall positive predictive values (PPVs) of biochemical and genetic NBS were 79.95% and 47.57%, respectively. A total of 15 variants were identified, with the six most common variants being c.1388G > A, c.1376G > T, c.95A > G, c.871G > A, c.1024C > T and c.392G > T (94.2%). The activity of G6PD was correlated with the type and WHO classification of variants.

**Conclusion:**

This study highlighted that combined screening could enhance the efficiency of current NBS for diagnosing G6PD deficiency. The prevalence, variant spectrum and allele frequency of G6PD deficiency vary across different regions. Our data provide valuable references for clinical practice and optimization of future screening strategies for G6PD deficiency.

## 1. Introduction

Glucose-6-phosphate dehydrogenase (G6PD) deficiency is the most prevalent human enzyme disorder, affecting more than 400 million people, globally, with an estimated prevalence of 4.9% [1]. It has a wide geographical distribution and is most commonly found in Africa, Asia, the Mediterranean region, and the Middle East [2]. G6PD deficiency is also prevalent in China and is more common in South China than in North China [3]. A recent study that screened 1,764,299 neonates between 2013–2017 reported an overall prevalence of G6PD deficiency in China of 0.77%, with the prevalence in South China being 0.95% and in North China, 0.03% [4].

G6PD deficiency is caused by loss-of-function variants in the *G6PD* gene, which is located on chromosome X at Xq28, contains 13 exons and 12 introns, and follows an X-linked pattern of inheritance [5, 6]. Being X-linked, males may be either hemizygous normal or hemizygous deficient, while females may be either homozygous normal, homozygous deficient or heterozygous [7]. According to the World Health Organization (WHO), *G6PD* variants can be categorized into five classes (Class I to V) based on the level of enzyme activity in erythrocytes and the clinical manifestations [8].

Although the majority of individuals with G6PD deficiency are asymptomatic throughout their lives, severe cases can result in severe hyperbilirubinemia and bilirubin encephalopathy (kernicterus) in the neonatal period, or acute non-immune hemolysis, triggered by fava beans ingestion, oxidative drugs, or exposure to an exogenous hemolytic trigger, such as bacterial or viral infections [1]. Early detection, intervention with phototherapy, and avoiding triggers are the most effective ways of managing this disorder.

NBS for G6PD deficiency is a simple, cost-effective strategy for identifying at-risk newborns. The WHO recommends that population screening of all newborns should be conducted in areas where the prevalence of G6PD deficiency is 3% to 5% or higher in males [8].

Since the 1980s, NBS for G6PD deficiency has been conducted in many regions of China. The routine screening assays are based on the semi-quantitative G6PD activity by fluorescence testing. Confirmatory tests are performed by determining absolute values of the G6PD activity or ratio of G6PD to 6-phosphogluconate dehydrogenase (6PGD) in erythrocytes [9, 10]. The semi-quantitative screening method is simple, rapid, sensitive, and inexpensive and can accurately identify hemizygous males and homozygous females [11]. However, detecting *G6PD* female heterozygotes using current biochemical assays remains a challenge, and molecular analysis may provide an alternative method of detection. Several molecular methods for detecting *G6PD* variant have been reported, including restriction fragment length polymorphism analysis [12], high-resolution melting curve assay [13], multiplex SNaPshot assay [14], Sanger sequencing [15], *etc*. However, these methods have low throughput, are time consuming, or both. Moreover, individuals with rare variants that are not included in a particular variant screening panel may be missed.

With the advent of next-generation sequencing technology and the decrease in cost, it has been deemed an innovative methodology for NBS [16–22]. However, it has been implemented as a second-tier test for high-risk infants. In addition, most of these studies used whole genome or whole exome sequencing, which cannot be universally applied in NBS because of its high cost. Moreover, there is no study on the outcomes of application of combined genetic and biochemical NBS for G6PD deficiency in parallel.

In 2021, a prospective multicenter study on genetic NBS as a first-tier screening test was conducted in China, with the participation of eight NBS centers, applying gene panel sequencing of 142 genes, including *G6PD* [23]. This study aimed to assess the outcomes of application of combined genetic and biochemical NBS for G6PD deficiency, and to investigate the molecular epidemiological characteristics, variant spectrum, and genotype-phenotype correlation of G6PD deficiency in China. To the best of our knowledge, this is the first study to evaluate outcomes of prospective application of combined genetic and biochemical NBS for G6PD deficiency in a general newborn population.

## 2. Material and methods

### 2.1 Subjects

A total of 29,601 newborns (15,365 males and 14,236 females) were prospectively recruited from eight NBS centers across eight hospitals in China from 21 February 2021 to 30 December 2021. The eight NBS centers were selected to represent different regions. From south to north, the participating hospitals were Hainan Woman and Children’s Medical Center, Guangzhou Woman and Children’s Medical Center, the First People’s Hospital of Yunnan Province, Chongqing Health Center for Women and Children, Xinhua Hospital affiliated to Shanghai Jiaotong University School of Medicine (located in South China), and Jinan Maternity and Child Care Hospital, Shijiazhuang Maternal and Child Health Care Hospital, Inner Mongolia Maternity and Child Health Care Hospital (located in North China).

The exclusion criteria included: (1) incomplete medical records (2) blood transfusion. This study was approved by the Ethical Committee of the participating hospitals, and written informed consent from parents was obtained before dried blood spot (DBS) collection.

### 2.2 Newborn screening for G6PD deficiency

DBS specimens were collected between 48 hours to seven days after birth and were tested for biochemical and genetic NBS simultaneously.

Biochemical NBS was performed in NBS centers using a fluorometric assay. Infants with the G6PD activity below a specified cut-off value (S 1) were recalled for confirmatory testing. G6PD deficiency was diagnosed when the enzyme activity of G6PD was less than 1700 U/L (normal range: 1700–4000 U/L) or when the ratio of G6PD/6PGD was less than 1.1 in erythrocytes (normal range: 1.1–2.5).

Genetic NBS was performed in Beijing Genomics Institute using a targeted gene panel sequencing test for 142 genes associated with 128 inherited disorders, including *G6PD*. The method was described previously [23]. Variants were classified and interpreted following the American College of Medical Genetics and Genomics and the Association for Molecular Pathology (ACMG/AMP) guidelines [24]. Only variants classified as pathogenic or likely pathogenic variants were reported. The preliminary genetic results were obtained within 14 days after receiving the DBS specimens. Infants with genetic NBS positive results were followed up through physical recall or telephone call.

The diagnostic criteria for G6PD deficiency are as follows: positive genetic NBS results with the enzyme activity of G6PD in DBS less than the cut-off value of the laboratory, or with positive results in biochemical confirmatory testing. Heterozygous females with negative biochemical results were identified as carriers.

### 2.3 Statistical analysis

Quantitative data were expressed as median with interquartile range. Statistical analyses were performed with SPSS version 19.0 (IBM, Chicago, Illinois, USA). The Fisher exact test or Chi squared was performed for the comparison of proportions. The t-test, Mann–Whitney U-test and Kruskal–Wallis test were used for parametric and non-parametric comparisons. A *p*-value of less than 0.05 was deemed statistically significant.

## 3. Results

### 3.1 Overview of biochemical and genetic NBS for G6PD deficiency

Of the 26,368 newborns with biochemical NBS results, 404 (319 males and 85 females) were initially screened positive, yielding a screening positive rate of 1.53% (404/26,368). Among these positive cases, 323 (276 males and 47 females) were diagnosed with G6PD deficiency. The overall positive predictive value (PPV) of biochemical NBS was 79.95% (86.52% for male, and 55.29% for female, *p* < 0.001) (Table 1).

**Table 1:** Epidemiological characteristics and comparison of biochemical NBS with genetic NBS for G6PD deficiency between South and North China.

Region		Enrolled subjects (n)			Biochemical NBS				Genetic NBS							
					confirmed subjects (n)		PPV (%)		confirmed subjects (n)		PPV (%)		Prevalence (%)			Carrier rate (%)
		M	F	M		F	PPV(M)	PPV(F)	M	F	PPV(M)	PPV(F)	M	F	Total	
South China	HN	520	457	49		18	85.96	75.00	49	18	100.00	30.00	9.42	3.94	6.86	9.19
	GZ	2558	2255	160		25	98.77	100.00	169	25	99.41	8.74	6.61	1.11	4.03	11.57
	YN	1538	1468	35		2	87.5	28.57	36	2	100.00	6.45	2.34	0.14	1.26	1.98
	CQ	1500	1488	11		1	100.00	100.00	11	1	100.00	11.11	0.73	0.07	0.37	0.54
	SH	2458	2430	11		1	100.00	50.00	11	1	100.00	6.25	0.45	0.04	0.25	0.62
North China	JN	2566	2231	9		0	24.32	NA	9	0	100.00	NA	0.35	NA	0.19	0.13
	SJZ	2533	2366	1		0	100.00	NA	1	0	100.00	NA	0.04	NA	0.02	0.25
	IM*	1692	1541	NA		NA	NA	NA	0	0	NA	NA	NA	NA	NA	0.13
**Total**		**15365**	**14236**	**276**		**47**	**86.52**	**55.29**	**286**	**47**	**99.65**	**11.38**	**1.86**	**0.33**	**1.12**	**2.57**

HN, GZ, YN, CQ, SH, JN, SJZ and IM stand for Hainan, Guangzhou, Yunnnan, Congqing, Shanghai, Jinan, Shijiazhuang and inner Mongolia, respectively. G6PD: Glucose-6-phosphate dehydrogenase; M: Male; F: Female; NA: not applicable; NBS: newborn screening; PPV: positive predictive value.

*: The biochemical NBS testing on G6PD deficiency in Inner Mongolia had not been established during the study period.

Genetic NBS identified *G6PD* variants in 701 cases (288 males and 413 females), yielding a variant detection rate of 2.37% (701/29,601). Among the 288 males with G6PD variants, 276 with positive biochemical NBS results were directly diagnosed with G6PD deficiency. Biochemical NBS was negative in 12 cases, among which 10 cases were recalled and were confirmed by biochemical confirmatory testing (biochemical false negatives) (Table 2). One was lost to follow-up and the other was diagnosed with Klinefelter syndrome (XXY). The activity of G6PD in the case with XXY to be 6.1 U/gHb (normal range: ≥2.6U/gHb) in DBS and the ratio of G6PD/6PGD in erythrocytes was 1.17. Among the 413 females with variants (366 carriers), 47 with positive biochemical NBS results were directly diagnosed with G6PD deficiency, including 18 with biallelic variants and 29 with heterozygous variants. The remaining 366 biochemical negative cases were classified as female carriers. The overall PPV of genetic NBS was 47.57% (99.65% for male, and 11.38% for female, *p* < 0.001) (Table 1).

**Table 2:** G6PD deficient patients undetected by biochemical NBS but confirmed by confirmatory testing.

NO	Region	Sex	Gestation week	Weight (g)	G6PD gene variant	WHO Class	Initial G6PD screening results (U/gHb)*	Confirmatory test results
P1	GZ	male	39	3640	c.392G>T	III	3.0	G6PD/6PGD = 0.35 (normal range: 1.1–2.5)
P2	GZ	male	39+4	3030	c.392G>T	III	2.7	G6PD/6PGD = 0.28 (normal range: 1.1–2.5)
P3	GZ	male	37	3850	c.392G>T	III	2.8	Specific data was unknown. The patient was followed by phone interview
P4	GZ	male	38+6	2780	c.392G>T	III	3.3	Specific data was unknown. The patient was followed by phone interview
P5	GZ	male	39+2	3080	c.392G>T	III	3.4	Specific data was unknown. The patient was followed by phone interview
P6	GZ	male	39	3500	c.392G>T	III	3.4	Specific data was unknown. The patient was followed by phone interview
P7	GZ	male	37+4	2360	c.1024C>T	III	2.8	G6PD/6PGD = 0.33 (normal range: 1.1–2.5)
P8	YN	male	39	3600	c.1024C>T	III	3.0	G6PD/6PGD = 0.2 (normal range: 1.1–2.5)
P9	GZ	male	38	2620	c.1388G>A	II	2.7	Specific data was unknown. The patient was followed by phone interview
P10	GZ	male	39+1	3480g	c.1388G>A	II	5.9	G6PD/6PGD = 0.9 (normal range: 1.1–2.5)

G6PD: Glucose-6-phosphate dehydrogenase; NBS: newborn screening; 6PGD: 6-Phosphogluconate Dehydrogenase; GZ: Guangzhou; YN: Yunnnan

*The cut-off value was 2.6 U/gHb.

Overall, the biochemical NBS for G6PD deficiency had a sensitivity of 97%, whereas genetic NBS had a sensitivity of 100%.

### 3.2 The prevalence of G6PD deficiency

A total of 333 patients (286 males and 47 females, 6.1:1) were diagnosed with G6PD deficiency, resulting in an overall prevalence of 1.12% (333/29,601), 1.86% (286/15,365) in male and 0.33% (47/14,236) in female. The overall prevalence of G6PD deficiency ranged from 0% to 6.86% in eight regions. There was a significant difference in prevalence of G6PD deficiency between South China and North China (1.94% *v*. 0.08%, *p* < 0.001). The overall prevalence of female carriers was 2.57% (366/14,236), with rates ranging from 0.13% to 11.57% (Table 1).

### 3.3 Variant spectrum, allele frequency and geographic distribution of *G6PD* variants in China

Among the 701 cases with *G6PD* variants, 288 individuals carried hemizygous variants, five carried homozygous variants, 13 carried compound heterozygous variants and 395 carried heterozygous variants. A total of 15 different variants were identified, including 14 reported pathogenic variants and one novel likely pathogenic variant (c.661_662delTT). The novel variant introduced frameshift and generated a premature termination signal. This variant was absent from public databases of 1000 genomes, dbSNP, Human Gene Mutation, and gnomAD. Together, it was classed as likely to be pathogenic (PVS1+ PM2), according to ACMG standards and guidelines for the interpretation of sequence variants.

The six most common variants observed were c.1388G > A, c.1376G > T, c.95A > G, c.871G > A, c.1024C > T, and c.392G > T, which accounted for 94.2% of the identified alleles. These common variants were shared by individuals from Hainan and Guangzhou. The c.487G > A variant was found to be common in Yunnan. In addition, rare variants, including c.661_662delTT, c.844G > C, and c.973G > A, were exclusively identified in newborns from North China (Table 3).

**Table 3:** The spectrum, allele frequencies, and geographic distribution of *G6PD* variants in China.

No.	G6PD gene variants	Exon	WHOClass	Frequency (n, %)	HN	GZ		YN	CQ	SH	JN	SJZ	IM
					(n = 111)	(n = 471)		(n = 68)	(n = 20)	(n = 27)	(n = 12)	(n = 7)	(n = 3)
1	c.1388G>A	12	II	229 (31.8%)	22.5%	37.8%		17.6%		6	3	4	1
2	c.1376G>T	12	II	220 (30.6%)	62.2%	27.0%		5.9%	6	10	2	1	1
3	c.95A>G	2	II	82 (11.4%)	7.2%	14.0%		5.9%	2	2			
4	c.871G>A	9	II	56 (7.8%)	4.5%	9.3%		5.9%	1	2			
5	c.1024C>T	9	III	55 (7.6%)	2.7%	5.1%		17.6%	6	6	4		
6	c.392G>T	5	III	35 (4.9%)	0.9%	5.3%		7.4%	2	1	1		
7	c.487G>A	6	III	23 (3.2%)				30.9%	1		1		
8	c.517T>C	6	II	6 (0.8%)		0.8%		1.5%	1				
9	c.592C>T	6	II	5 (0.7%)		0.4%		2.9%				1	
10	c.406C>T^#^	5	II	3 (0.4%)				2.9%	1				
11	c.209A>G^#^	4	III	1 (0.1%)				1.5%					
12	c.563C>T	6	II	1 (0.1%)		0.2%							
13	c.661_662delTT^#^	7	NR	1 (0.1%)									1
14	c.844G>C	8	III	1 (0.1%)							1		
15	c.973G>A	9	NR	1 (0.1%)								1	
Total				719 (100%)	100.0%	100.0%	100.0%		20	27	12	7	3

G6PD: Glucose-6-phosphate dehydrogenase; NBS: newborn screening; NR: Class not reported

#:Novel variant; HN, GZ, YN, CQ, SH, JN, SJZ and IM stand for Hainan, Guangzhou, Yunnnan, Congqing, Shanghai, Jinan, Shijiazhuang and inner Mongolia, respectively. The allele distribution of each province was expressed as percentage (n≥30) or number of alleles (n<30).

### 3.4 Relationship between G6PD genotype and the G6PD activity in DBS specimens in Guangzhou, China

A total of 194 patients born in Guangzhou were diagnosed with G6PD deficiency, including 169 hemizygous males, five homozygous, 10 compound heterozygous and 10 heterozygous females. There was no significant difference in the G6PD activity between the male-hemizygous and female-biallelic variant groups [1.2 (0.8) *v*. 1.2 (0.6) U/g Hb, *p* = 0.792]. There were significant differences in the G6PD activity between the male-hemizygous and female-heterozygous groups [1.2 (0.8) *v*. 2.3 (0.5) U/g Hb, *p* < 0.001)] and between the female-biallelic variant and female-heterozygous groups [1.2 (0.6) U/g *v*. 2.3 (0.5) U/g Hb, *p* < 0.001)].

Among the 169 male patients with *G6PD* variants, nine were undetected by biochemical NBS, among which, six (66.7%) harbored the c.392G > T variant (Class III), two (22.2%) harbored the c.1024C > T variant (Class III), and 1 (11.1%) harbored the c.1388G > A variant (Class II) (Table 2). Class II variants (c.1388G > A, c.1376G > T, c.95A > G, c.871G > A, and c.592C > T) composed the predominant pattern, accounting for 87.6% of cases. The G6PD activity in individuals with Class II variants was significantly lower than that of cases with Class III variants [1.1 (0.6) *v*. 2.2 (1.2) U/g Hb, *p* < 0.001)] (Table 4).

**Table 4:** Relationship between G6PD genotype and the enzyme activity in DBS specimens of G6PD-deficient males from Guangzhou, China.

Nucleotide change	Amino acid change	Class*	Frequency (n, %)	G6PD activity in NBS (U/gHb)
c.1388G>A	p.R463H	II	73 (43.2%)	1.2 (0.5)
c.1376G>T	p.R459L	II	33 (19.5%)	0.7 (0.4)
c.95A>G	p.H32R	II	26 (15.4%)	1.0 (0.5)
c.871G>A	p.V291M	II	15 (8.9%)	1.4 (0.7)
c.1024C>T	p.L342F	III	15 (8.9%)	1.9 (0.6)
c.392G>T	p.G131V	III	6 (3.6%)	3.2 (0.6)
c.592C>T	p.R198C	II	1 (0.6%)	0.3
Total			169 (100%)	1.2 (0.8)

G6PD: Glucose-6-phosphate dehydrogenase; DBS:dried blood spot; *WHO classification of G6PD variants; NBS: newborn screening; G6PD activity was showed as median with interquartile range.

## 4. Discussion

This prospective multicenter study, assessed the outcomes of application of combined genetic and biochemical NBS for G6PD deficiency, determined the prevalence of G6PD deficiency, and investigated the variant spectrum, and the genotype-phenotype correlations of G6PD deficiency. To the best of our knowledge, this is the first and largest prospective study to evaluate the outcomes of combined biochemical and genetic NBS for G6PD deficiency in the general newborn population. Our study provides valuable insights for optimizing future screening strategies for G6PD deficiency in clinical practice.

Comparison of biochemical and genetic NBS for G6PD deficiency showed that genetic NBS demonstrated a sensitivity of 100%, while the sensitivity of the biochemical NBS was 97%. The PPV of biochemical NBS varied in different regions, which might be attributed to the setting of positive cut-off values in different NBS centers. Moreover, the overall PPV of biochemical NBS was significantly higher than that of genetic NBS (79.95% *v*. 47.57%, *p* < 0.001); the overall PPV of genetic NBS for males was significantly higher than that for females (99.65% *v*. 11.38%, *p* < 0.001). These were mainly due to the fact that female with heterozygous variants were also considered as positive in genetic NBS in this study and most female heterozygotes exhibit normal G6PD activity due to skewed X-chromosome inactivation [7]. Additionally, our data showed a high concordance between the biochemical NBS results of hemizygous males and females with biallelic variants and their corresponding genetic NBS results. These individuals could be rapidly diagnosed with G6PD deficiency and receive telephonic genetic counselling and education, which would eliminate the need to resubmit whole blood samples for diagnostic confirmation. However, it is worth noting that in the absence of biochemical results, genetic NBS was unable to differentiate between female carriers, and those with G6PD deficiency from X-inactivation. Together, these data indicate that the current biochemical NBS method was reliable as a first-tier NBS test in South China, and genetic NBS had the potential to supplement biochemical NBS in detection capability but cannot replace it as a universal NBS program. Overall, our study highlights the importance of combining biochemical and genetic NBS for G6PD deficiency, particularly in identifying male patients with mild variants and detecting female carriers. These findings have significant implications for optimizing screening strategies and improving clinical management of G6PD deficiency and will be a guiding role in rational drug use in the patients with G6PD deficiency.

The data revealed significant regional variations in the prevalence of G6PD deficiency, the spectrum of variants, and the allele frequency of *G6PD* in China. The prevalence of G6PD deficiency was higher in South China compared to North China, which aligns with the findings of a previous study [3]. Historically, South China was endemic for malaria: since G6PD deficiency imparts a selective advantage against malarial infection, the distribution of G6PD deficiency is closely related to the prevalence of malaria. Our epidemiological investigation revealed that the distribution of G6PD deficiency was geographically concordant with the area of historical malaria prevalence in China.

The six variants c.1388G > A, c.1376G > T, c.95A > G, c.871G > A, c.1024C > T, and c.392G > T were the most common, accounting for 94.2% of cases. These findings were consistent with earlier observations from China [4] and our previous research [11]. However, they differed from the predominant variants reported in India, in which c.131C > G, c.563C > T, and c.949 G > A were the most common variations (81.3%) [25]. These variations could be attribute to difference in the demographic composition in different populations.

Limited research has been conducted on the genotype-phenotype association of G6PD deficiency [26–29]. In our study, the G6PD activity was found to be correlated with the type and the WHO classification of variants. Hemizygous males, homozygous and compound-heterozygous females had a much lower G6PD activity than heterozygous females, which were consistent with a previous study [26], and the G6PD activity of Class II variants was significantly lower than that of cases with Class III variants. It is important to highlight that biochemical NBS missed some male patients with mild variants, especially those carrying the c.392G > T variant (Class III). Several factors may contribute to the false-negative results, including the setting of the cut-off value, differences in reticulocyte count, mean corpuscular hemoglobin values in the test sample, and sex chromosome aneuploidy of individuals [7]. Several patients with mild variants and false-negative results in our study had enzyme activity near the cut-off value. With some *G6PD* variants, young red cells and particularly reticulocytes (as seen in a hemolytic episode) have a much higher G6PD activity than mature red cells. The mean corpuscular hemoglobin (pg) of the test sample should be taken into account, as very low values (as seen in thalassemia and iron deficiency) will overestimate the activity of G6PD. Male individuals with Klinefelter syndrome (XXY) may have intermediate levels similar to those of heterozygous females due to skewed X-chromosome inactivation. In our study, the case with XXY also had an intermediate level with a G6PD/6PGD ratio of 1.17.

Some limitations should be noted when reviewing the present study. Firstly, biochemical confirmatory testing was not performed in all female cases with *G6PD* variants and thus false negative tests on the biochemical assay may have been missed. Secondly, we did not evaluate the cost-effectiveness of a gene panel as a first-tier screening test.

In summary, our study has shown that the combination of biochemical and genetic NBS can improve the efficiency of NBS for diagnosing G6PD deficiency. This study effectively characterized the prevalence, variant spectrum, and genotype-phenotype correlation of G6PD deficiency in China. Our data will provide valuable epidemiological and clinical practice references for the standardization of future management of G6PD deficiency.

## Supporting information

S1 Table Method and cut-off value of biochemical NBS for G6PD deficiency in 8 NBS centers. (DOCX)
