# Supplementary material for: Molecular epidemiological characteristics, variant spectrum and genotype-phenotype correlation of glucose-6-phosphate dehydrogenase deficiency in China: A population-based multicenter study using newborn screening
Source: PLoS One. 2024 Oct 22;19(10):e0310517. doi: 10.1371/journal.pone.0310517 (PMC11495603; doi:10.1371/journal.pone.0310517)
Supplement: S1 Table — (DOCX) [file pone.0310517.s001.docx]

**S1 Table Method and cut-off value of biochemical NBS for G6PD deficiency in 7 NBS centers.**

| Region | Method | cutoff-value | Assay Kit | Instrument |
| --- | --- | --- | --- | --- |
| HN | fluorometric method | 26 U/dL | Perkin Elmer | Automatic fluorescence immunoanalyzer |
| GZ | quantitative fluorescence assay | 2.6U/gHb | Guangzhou Fenghua Biology | Perkin Elmer 1420 fluorescence analyzer |
| YN | quantitative fluorescence assay | 2.6U/gHb | Finland Wallac Oy | WALLAC-1420 fluorescence reader, Finland |
| CQ | quantitative fluorescence assay | 5U/gHb | Thermo Finnpipette | Guangzhou Lambo F301 fluorescence analyzer |
| SH | quantitative fluorescence assay | 20 U/dL | Perkin Elmer | Automatic fluorescence immunoanalyzer |
| JN | quantitative fluorescence assay | 27 U/dL | Perkin Elmer | Automatic fluorescence immunoanalyzer |
| SJZ | quantitative fluorescence assay | 3.5U/gHb | Thermo Finnpipette | Perkin Elmer 1420 fluorescence analyzer |

G6PD: Glucose-6-phosphate dehydrogenase; NBS: newborn screening; HN, GZ, YN, CQ, SH, JN, SJZ and IM stand for Hainan, Guangzhou, Yunnnan, Congqing, Shanghai, Jinan, Shijiazhuang and inner Mongolia, respectively.
